# Cell Surface Vimentin Is an Attachment Factor That Facilitates Equine Arteritis Virus Infection In Vitro

**DOI:** 10.3390/v18010113

**Published:** 2026-01-15

**Authors:** Côme J. Thieulent, Sanjay Sarkar, Mariano Carossino, Mouli Bhowmik, Haining Zhu, Udeni B. R. Balasuriya

**Affiliations:** 1 Department of Pathobiological Sciences, School of Veterinary Medicine, Louisiana State University, Baton Rouge, LA 70803, USA; cthieulent@lsu.edu (C.J.T.); mcarossino1@lsu.edu (M.C.); 2 Infectious Disease Research, Southern Research, 2000 9th Avenue S, Birmingham, AL 35205, USA; ssarkar@southernresearch.org; 3 Louisiana Animal Disease Diagnostic Laboratory, School of Veterinary Medicine, Louisiana State University, Baton Rouge, LA 70803, USA; 4 Department of Pediatrics, University of Alabama at Birmingham Heersink School of Medicine, Birmingham, LA 53233, USA; moulibhowmik@uabmc.edu; 5 Department of Pharmacology and Toxicology, R Ken Coit College of Pharmacy, University of Arizona, Skaggs Pharmaceutical Sciences Center, 1703 E. Mabel Street, Tucson, AZ 85721, USA; haining@arizona.edu

**Keywords:** equine arteritis virus, equine viral arteritis, virus receptor, vimentin, equine CXCL16

## Abstract

Our laboratory identified the susceptible allelic variant of equine CXCL16 protein (EqCXCL16S) as an entry receptor for equine arteritis virus (EAV). However, EAV has a broad host cell tropism and infects cells that lack EqCXCL16S. Thus, we hypothesized that EAV interacts with other host cell protein(s) that facilitate EAV infection. A virus overlay protein-binding assay in combination with a Far-Western blot from EAV-susceptible equine pulmonary artery endothelial cells (EECs) and equine dermal fibroblasts (E. Derm) identified a 57 kDa protein, present in the membrane fraction of the protein lysate, as a possible EAV-binding protein. Subsequent LC-MS/MS analysis identified this 57 kDa protein as vimentin. Screening of different mammalian cell lines has shown that only cells expressing vimentin are susceptible to EAV infection. Pre-treatment of EECs with an anti-vimentin polyclonal antibody and Withaferin A partially inhibit EAV infection. Finally, the overexpression of equine vimentin (EqVim) in HEK-293 cells increases their susceptibility to EAV infection. Overall, our data strongly indicate that EAV binds to the host cell protein equine vimentin, which actively participates in EAV infection, potentially serving as an attachment factor. The data suggest that EAV interacts with various host cell proteins to achieve its diverse cell tropism.

## 1. Introduction

Equine arteritis virus (EAV), recently renamed *Alphaarterivirus equid*, is a single-stranded, positive-sense RNA virus belonging to the genus *Alphaarterivirus*, subfamily *Equarterivirinae*, family *Arteriviridae*, order *Nidovirales* [1,2]. EAV was first isolated from the lung of a fetus during an abortion storm on a horse farm in Bucyrus, Ohio, in 1957 [3,4]. EAV is the causative agent of equine viral arteritis (EVA), a respiratory and reproductive disease with a worldwide prevalence, resulting in significant economic losses to the equine industry [5]. EAV infections are mostly subclinical, but they can be associated with a wide range of clinical signs, including respiratory distress, flu-like illness, dependent edema, conjunctivitis, periorbital or supraorbital edema, urticaria, and leukopenia [6,7,8,9,10,11,12,13]. Infection of pregnant mares may result in abortion or birth of congenitally infected foals, developing rapidly fatal bronchointerstitial pneumonia or pneumoenteric syndrome [14,15]. EAV is transmitted by the respiratory route, via infective aerosolized secretions, and by the venereal route, via infective semen derived from infected stallions [5]. Following infection, EAV establishes long-term persistent infection (LTPI) in the stallion’s reproductive tract, resulting in the continuous shedding of infectious virus in the semen [13,16]. Our previous studies demonstrated that EAV infects and persists in the ampullae of the stallion reproductive tract, exhibiting a specific tropism for vimentin-positive stromal cells within the lamina propria, as well as for CD8^+^ T cells and CD21^+^ B lymphocytes [13], despite the presence of strong serological, mucosal, and seminal plasma immune responses [17]. EAV also infects monocytes, macrophages, and a small subpopulation of CD3^+^ T lymphocytes in equine peripheral blood mononuclear cells (PBMCs) in vitro [18,19,20]. We have also demonstrated that CD3^+^ T cell infection is mediated by a specific allele (*CXCL16^S^*) of the equine C-X-C motif chemokine ligand 16 (EqCXCL16) protein that renders these cells susceptible to EAV infection in vitro [21]. In contrast, CD3^+^ T cells from horses homozygous for the resistant allele of the EqCXCL16 (*CXCL16^r^*) are resistant to EAV infection in vitro [22]. Furthermore, we have demonstrated that stable transfection of HEK-293 cells with equine *CXCL16^S^* increases susceptibility to EAV infection, confirming that EqCXCL16S can act as a cell entry receptor for EAV [21]. Moreover, EAV also preferentially infects subpopulations of horse CD14+ monocytes expressing EqCXCL16S, and that infection of these cells is significantly reduced by pretreatment with anti-EqCXCL16 polyclonal antibodies raised in guinea pig [22]. Interestingly, a population of CD14^+^ cells derived from horses homozygous for the *CXCL16^r^* allele is also susceptible to EAV infection, indicating that other cellular receptors are involved in virus entry. EqCXCL16 differs substantially from CXCL16 orthologues in other species [22], indicating that non-equine CXCL16 sequences are unlikely to function as entry receptors. However, it is well-documented that a wide variety of continuous cell lines from different mammalian species other than equids (which obviously do not express equine CXCL16) support EAV replication, including African green monkey kidney cells (Vero and MA-104), baby hamster kidney fibroblasts (BHK-21), normal rabbit kidney epithelial (RK-13) cells, HeLa cells, and mouse connective tissue cells (L-M) [9,23,24,25,26,27,28,29,30,31]. In addition, it has been demonstrated that heparin treatment partially inhibits EAV infection of RK-13 cells, suggesting that heparin may play a role in EAV binding; however, it is not the only critical factor [32,33]. Recently, CD81 from equine and other species was identified as another cellular receptor for EAV in vitro [31]. These data suggest the involvement of other receptors(s) or cofactors(s) as determinants of EAV cell tropism. Therefore, the goal of this study was to identify additional receptor(s) or accessory cell proteins involved in EAV infection in vitro.

## 2. Materials and Methods

### 2.1. Cells

Equine pulmonary artery endothelial cells (EECs) [34,35,36] were propagated in Dulbecco’s modified Eagle’s medium (DMEM with 4.5 g/L glucose, sodium pyruvate, and L-glutamine; Corning, New-York, NY, USA) supplemented with 10% ferritin-supplemented bovine calf serum (Hyclone Laboratories LLC, Logan, UT, USA), 1 mM sodium pyruvate (Gibco, Waltham, MA, USA), 2 mM L-glutamine (Gibco), and 0.1 mM non-essential amino acids (NEAA; Gibco). Equine dermal fibroblasts (E. Derm, NBL-6 ATCC^®^ CCL-57, American Type Culture Collection [ATCC^®^], Manassas, VA, USA) and the human breast cancer cell line MCF7 (ATCC^®^ HTB-22) were propagated in Eagle’s minimal essential medium (EMEM; with Earle’s salts and L-glutamine; Corning) supplemented with 10% fetal bovine serum (FBS), 1 mM of sodium pyruvate and 0.1 mM NEAA. Baby hamster kidney (BHK-21, C-13, ATCC^®^ CCL-10), and high-passage rabbit kidney cells (HP-RK-13 [KY] P399-409, originally derived from ATCC CCL-37^TM^) were propagated in EMEM supplemented with 10% ferritin-supplemented bovine calf serum. The human colorectal adenocarcinoma cell line DLD-1 (ATCC^®^ CCL-221) was propagated in RPMI 1640 (Gibco) supplemented with 10% FBS and 2 mM L-glutamine. Human embryonic kidney cells (HEK-293, ATCC^®^ CRL-1573) were propagated in DMEM supplemented with 10% FBS and 2 mM L-glutamine. All media contained 100 IU/mL penicillin, 100 µg/mL streptomycin (Gibco), and 0.25 µg/mL amphotericin B (Gibco). Cells were cultivated in a humidified incubator at 37 °C and 5% CO_2_.

### 2.2. Viruses

Two strains of EAV, the virulent Bucyrus strain (VBS; ATCC ^®^ VR-796) [3,4] and the recombinant EAV VBS strain expressing mCherry (EAV sVBSmCherry) [37], were used in this study. Both viruses were used at their second passage on EECs, and high-titer stocks were generated as previously described [22,35].

### 2.3. Antibodies and Chemical Compounds

Mouse monoclonal antibodies specific to EAV GP5, N and nsp1 (6D10, 3E2, and 12A4, respectively) have been described previously [38,39]. Goat anti-rabbit IgG (H + L)-HRP and goat anti-mouse IgG (H + L)-HRP were purchased from Cell Signaling Technology (Danvers, MA, USA) and Southern Biotech (Birmingham, AL, USA). Mouse anti-vimentin MAb (Clone V9) and chicken anti-vimentin polyclonal antibody (PA1-10003) were purchased from Invitrogen (Waltham, MA, USA). Normal chicken IgY isotype control (AB-101-C) was purchased from R&D Systems (Minneapolis, MN, USA). Goat anti-mouse IgG (H + L) conjugated to Alexa Fluor (AF) 488, Goat anti-mouse IgG (H + L) conjugated to AF 647 were purchased from Life Technologies (Grand Island, NY, USA). Withaferin A (HY-N2065; MedChemExpress, Monmouth Junction, NJ, USA) was dissolved in DMSO (Sigma-Aldrich, Saint-Louis, MO, USA) at 10 mM and stored at −20 °C until used.

### 2.4. Virus Overlay Protein Binding Assay and Far-Western Blot

One hundred micrograms of total protein lysate from EECs and E. Derm cells or 30 μg of membrane or cytoplasmic protein fractions from EECs were separated in 12% SDS-PAGE and transferred onto a polyvinylidene fluoride (PVDF; Bio-Rad, Hercules, CA, USA) membrane for Far-Western blot (Far-WB) analysis following a modification of the published protocol [21,22]. The bound proteins were then denatured and gradually renatured on the membrane by sequential incubation with 6 M, 3 M, 1 M, and 0.1 M guanidine-HCl in freshly prepared AC buffer (100 mM NaCl, 20 mM TRIS [pH 7.5], 10% glycerol, 0.5 mM Ethylenediaminetetraacetic Acid [EDTA], 0.1% Tween-20, 2% non-fat dry milk, and 5 mM DTT) for 30 min at room temperature or with only AC buffer in the absence of guanidine-HCl overnight at 4 °C. The membrane was blocked with 5% non-fat dry milk in TBS-T (0.1% Tween-20) and overlaid with purified wild-type EAV VBS (15 μg/mL) and incubated overnight at 4 °C. The next day, the membrane was washed vigorously three times for 10 min and incubated with the mouse MAb (6D10) directed against EAV GP5 envelope glycoprotein. MAb binding was detected using a goat anti-mouse IgG conjugated to HRP and the ECL-detection system using SuperSignal^®^ West Pico chemiluminescent substrate (Thermo Fisher Scientific, Waltham, MA, USA).

### 2.5. Separation of Cytoplasmic and Membrane Protein Fractions

EECs were washed in ice-cold 1× phosphate-buffered saline (PBS), scraped, and then pelleted at 300 × g for 5 min. Cytoplasmic and membrane protein extracts were prepared using the Mem-PER^TM^ Plus Membrane Protein Extraction Kit (Thermo Fisher Scientific) following the manufacturer’s protocol. Briefly, the cell pellet was washed twice with Cell Wash solution and resuspended in permeabilization buffer, vortexed to get a homogeneous cell suspension. After incubating at 4 °C for 10 min, cells were centrifuged at 16,000× *g* for 15 min to collect the supernatants containing cytosolic proteins. The pellet was resuspended in solubilization buffer and incubated at 4 °C for 30 min with constant mixing, then pelleted again at 16,000 × g for 15 min at 4 °C. The supernatants containing cytosolic proteins and the membrane and membrane-associated proteins were stored at −80 °C until used.

### 2.6. Protein Trypsin Digestion and LC-MS/MS Analysis

EECs were solubilized, and integral membrane proteins and membrane-associated proteins were enriched and separated from cytoplasmic proteins using the Mem-PER^TM^ Plus Membrane Protein Extraction Kit (Thermo Fisher Scientific) as described above. One hundred micrograms of membrane and cytoplasmic proteins were subjected to 12% SDS-PAGE separation. The protein gel band at ~57 kDa was excised and subjected to dithiothreitol reduction, iodoacetamide alkylation, and in-gel trypsin digestion using a standard protocol, and the tryptic peptides were subjected to shotgun proteomics analysis as previously described [40]. The resulting tryptic peptides were extracted, concentrated to 15 µL using a SpeedVac, and 3 µL was injected for nano-liquid chromatography-tandem mass spectrometry (LC-MS/MS) analysis using an LTQ-Orbitrap mass spectrometer (Thermo Fisher Scientific) coupled with an Eksigent Nanoflex cHiPLC™ system (Eksigent, Dublin, CA, USA) through a nano-electrospray ionization source. The peptide samples were separated with a reversed phase cHiPLC column (75 μm × 150 mm) at a flow rate of 300 nL/min. Mobile phase A was water with 0.1% (*v*/*v*) formic acid, while B was acetonitrile with 0.1% (*v*/*v*) formic acid. A 50 min gradient condition was applied: initial 3% mobile phase B was increased linearly to 50% in 24 min and further to 85% and 95% for 5 min each before it was decreased to 3% and re-equilibrated. The mass analysis method consisted of one segment with eight scan events. The first scan event was an Orbitrap MS scan (100–1600 m/z) with 60,000 resolutions for parent ions, followed by data-dependent MS/MS for fragmentation of the seven most intense ions with the collision-induced dissociation (CID) method. The LC-MS/MS data were submitted to a local mascot server for MS/MS protein identification via Proteome Discoverer^TM^ (version 1.3, Thermo Fisher Scientific) against *Equus caballus* of the NCBI database (NCBI: txid9796). Parameters used in the MASCOT MS/MS ion search were trypsin digest with a maximum of two miscleavages, cysteine carbamidomethylation, methionine oxidation, a maximum of 10 ppm MS error tolerance, and a maximum of 0.8 Da MS/MS error tolerance. A decoy database was built and searched. Filter settings that determine false discovery rates (FDR) are used to distribute the confidence indicators for the peptide matches. Peptide matches that pass the filter associated with the strict FDR (with a target setting of 0.01) are assigned as high confidence. For MS/MS ion search, proteins with two or more high-confidence peptides were considered unambiguous identifications without manual inspection. Proteins identified with one high-confidence peptide were manually inspected and confirmed.

### 2.7. Time-Course Infection

Approximately 3 × 10^5^ cells (EECs, E. Derm, HP-RK-13, BHK-21, HEK-293, DLD-1, and MCF7) were seeded in 24-well plates and incubated at 37 °C and 5% CO_2_ in a humidified incubator. After 24 h, the supernatant was removed, and cells were infected with EAV sVBSmCherry at a multiplicity of infection (MOI) of 1 for 1 h at 37 °C. After three washing steps with 1× PBS, 500 μL of medium was added, and the plates were returned to the incubator. The plates were frozen and thawed three times at 12, 24, and 48 h post-infection (hpi), and the cell lysate was collected for virus titration. A total of 3 × 10^5^ HEK-293, HEK_CTL, and HEK_EqVIM seeded in 24-well plates and infected with EAV sVBSmCherry were placed in an IncuCyte^®^ Live Cell Analysis System (Sartorius, Göttingen, Germany), and the phase area and red area were recorded every six hours. The percentage of red area divided by the phase area was calculated using the IncuCyte^®^ 2022B Rev2 software.

### 2.8. Virus Titer Determination by Plaque Assay

Cell lysates were ten-fold diluted and inoculated on HP-RK-13 cells in a 12-well plate format. After 1 h of adsorption at 37 °C, cells were overlaid with complete EMEM containing 0.75% carboxymethylcellulose (Sigma-Aldrich) and incubated for 96 h at 37 °C and 5% CO_2_ in a humidified incubator. Media was removed, cells were subsequently fixed and stained with 0.2% crystal violet solution (Sigma-Aldrich) in 10% neutral buffered formalin for 3 h, and subsequently rinsed with tap water. Plaques were counted, and viral titers were determined as PFU per ml.

### 2.9. Blocking Cell Surface Vimentin with Anti-Vimentin Polyclonal Antibody and Withaferin A

Approximately 3 × 10^5^ of EECs were seeded in 24-well plates and incubated at 37 °C and 5% CO_2_ in a humidified incubator. After 24 h, the supernatant was removed, and cells were pre-treated with 10, 20, 40, and 80 µg/mL of vimentin polyclonal antibody (PA1-10003) or with equivalent concentrations of an isotype control for 1 h at 37 °C, diluted in fresh medium without FBS. Another set of cells was also pre-treated with 0.25, 0.5, 1, and 2 µM of Withaferin A (WFA) or DMSO (Sigma-Aldrich) diluted in fresh medium without FBS for 4 h at 37 °C. After three washing steps with 1× PBS, the cells were infected with EAV sVBSmCherry at an MOI of 0.1 for 1 h at 37 °C. After three washing steps with 1× PBS, fresh complete medium was added, and cells were incubated at 37 °C for 24 h. The cytotoxicity effect of WFA (0.0625 µM to 32 µM) on EECs was determined using CellTiter-Glo^®^ (Promega, Madison, WI, USA) at 24 h treatment as previously described [41].

### 2.10. Establishment of Stable HEK Cells Expressing Equine Vimentin

A stable cell line expressing equine vimentin (EqVim) was generated as described previously [21], with minor modifications. Briefly, for expression of EqVim protein in HEK-293 cells, a codon-optimized full-length EqVim sequence (NCBI Reference Sequence: NM_001243145.1) was commercially synthesized and cloned into the pIRESpuro3 plasmid (Takara Bio USA, San Jose, CA, USA) and named pIRESpuro3_EqVIM. In this construct, both EqVIM and puromycin-resistance sequences are expressed under the human cytomegalovirus immediate-early promoter and are separated by an internal ribosome entry site (IRES), enabling the co-expression of the two proteins from a single messenger RNA. An empty pIRESpuro3 vector, containing only the puromycin-resistance sequences, was used as a negative control (pIRESpuro3_CTL). Plasmid stocks were prepared by the transformation of original plasmids into chemically competent DH5α *E. coli* (Thermo Fisher Scientific) by the standard heat shock protocol. Bacteria were grown overnight on sterile LB broth supplemented with 100 mg/mL ampicillin. Plasmids were purified using the Plasmid Plus Maxiprep Kit (Qiagen, Valencia, CA, USA). HEK-293 cells seeded in 6-well plates (2 × 10^6^ cells/well) were transfected with 3 μg of pIRESpuro3_EqVIM and pIRESpuro3_CTL using Lipofectamine 3000 (Life Technologies, Grand Island, NY, USA), in serum-free conditions, according to the manufacturer’s instructions. At 48 h post-transfection, the medium was replaced with fresh medium containing 4 µg/mL of puromycin (Gibco). Culture media was replaced every other day until only the puromycin-resistant colonies were selected. These puromycin-resistant cells were cloned by limiting dilution in 96-well plates and screened by IFA to identify clones expressing a high level of EqVim protein expression. All the experiments in HEK_EqVim cells were performed within passages 7 to 10.

### 2.11. Indirect Immunofluorescence Assay (IFA)

EECs, E. Derm, HEK293, DLD-1, and MCF7 were seeded in 24-well plates containing cover slips (VWR, Radnor, PA, USA). Cells were washed in cold 1× PBS (pH 7.4) and fixed in 4% paraformaldehyde (PFA) (Sigma Aldrich, St. Louis, Missouri, USA) for 30 min at room temperature (RT). Cells were then stained following our previously published protocol [42]. Briefly, the cells were then washed three times with cold 1× PBS containing 10 mM glycine (PBS-Glycine; Sigma Aldrich) and permeabilized with 0.2% Triton X-100 (Sigma Aldrich) diluted in 1× PBS for 10 min or without detergent when examination of surface staining was required. Cells were washed in PBS-glycine and blocked with 5% FBS for 30 min at RT before incubation with specific primary antibodies (vimentin MAb, Clone V9; 1:400 dilution; mouse-anti EAV nsp1, Clone 12A4, 1:800 dilution) for 1 h at 37 °C. Cells were washed and incubated with conjugated secondary antibodies for 40 min at RT. Hoechst 33,342 (Invitrogen) solution was added to each well for an additional 20 min for nuclear staining (final concentration: 2 µg/mL). After washing, the coverslip was mounted on a glass slide using Fluoromount-G^TM^ mounting medium (SouthernBiotech, Birmingham, AL, USA). Fluorescence was examined using an Olympus FV300 confocal microscope (Hachioji, Tokyo).

### 2.12. SDS PAGE and Western Immunoblotting

HP-RK13, EECs, E. Derm, BHK-21, HEK-293, DLD-1, and MCF7 were lysed in 1× RIPA lysis buffer (Santa Cruz Biotechnology, Dallas, TX, USA) containing Halt phosphatase inhibitor cocktails (Thermo Fisher Scientific) and 1 mM of Phenylmethanesulfonyl Fluoride (PMSF). After 10 min of incubation on ice, the protein lysates were collected and centrifuged at 14,000× *g* for 10 min at 4 °C, and the supernatant was collected and stored at −80 °C until used. The solubilized proteins were mixed with Pierce 5× Lane reducing marker sample buffer containing 100 mM dithiothreitol (DTT) (Thermo Fisher Scientific). Samples were resolved in SDS-polyacrylamide gel (5% stacking and 12% resolving) at 200 V for 30 to 45 min and then transferred onto a PVDF membrane at 25 V, 2.5 A for 7 min using the Trans-Blot Turbo Transfer System (Bio-Rad, Hercules, CA, USA). The membrane was blocked with EveryBlot Blocking Buffer (Bio-Rad) for 10 min at RT and incubated with primary antibodies (Abs), (mouse anti-vimentin MAb (Clone V9) [1:1000], rabbit anti-β-actin [1:1000], and mouse anti-EAV GP5 MAb [6D10, 1:2000] diluted in EveryBlot Blocking Buffer overnight at 4 °C. The following day, the membranes were washed with TBS-T (Bio-Rad) and then incubated with secondary antibodies (anti-rabbit, or anti-mouse IgG, as appropriate), conjugated with horseradish peroxidase (HRP, 1:3000) (Cell Signaling, Danvers, MA, USA), diluted in EveryBlot Blocking Buffer for 1 h at RT. The membranes were washed again, and antibody binding was visualized with a ChemiDoc Imaging System (Bio-Rad) using Clarity Western ECL Substrate (Bio-Rad). Relative protein expression was quantified by densitometric analysis of Western blot bands using ImageJ (version 1.54d) software. The intensity of each target protein band was normalized to that of the corresponding loading control (β-actin) to correct for variations in protein loading. The normalized values were then expressed relative to the control sample, which was set to 1, to determine the fold change in protein expression across samples.

### 2.13. Flow Cytometry Analysis

HP-RK13, EECs, E. Derm, BHK-21, HEK-293, DLD-1, and MCF7 were harvested and washed with Fluorescence-activated cell sorting (FACS) buffer (1× PBS containing 0.1% sodium azide (VWR) and 1% heat-inactivated sterile-filtered goat serum (EquiTech-Bio, Inc., Kerrville, TX, USA)). Subsequently, cells were fixed with 4% PFA and labeled for 30 min at 4 °C with mouse anti-vimentin MAb (Clone V9; 1:400 dilution in FACS buffer), washed three times with FACS buffer, and stained with goat anti-mouse IgG (H + L) conjugated to AF 488 (1:400 dilution in FACS buffer). After three additional washing steps, flow cytometry data were collected using a BD FACS Calibur™ cytometer with 10^5^ events and analyzed with FlowJo version 10 software (BD Biosciences).

### 2.14. Equine CXCL16 Genotyping Determination

A total of 5.0 × 10^6^ E. Derm and EECs were collected and subjected to DNA extraction using DNeasy Blood & Tissue Mini Kit (Qiagen, Valencia, CA, USA), following the manufacturer’s instructions. Equine CXCL16 genotype was determined using the custom TaqMan^®^ SNP Genotyping Assay developed by our laboratory [43].

### 2.15. Statistical Analysis

Statistical analyses were performed using GraphPad Prism v10.2.3 statistical analysis software (GraphPad, San Diego, CA, USA). Data with a normal distribution were analyzed using one-way or two-way ANOVA followed by Dunnett’s post hoc test, while non-normally distributed data were evaluated using the Friedman non-parametric test with Dunn’s *post hoc* test. Data are expressed as mean ± standard deviation (SD). * *p* ≤ 0.05, ** *p* ≤ 0.01, *** *p* ≤ 0.001.

## 3. Results

### 3.1. A 57 kDa Host Cell Membrane Protein Specifically Interacts with EAV

**Figure 1:**
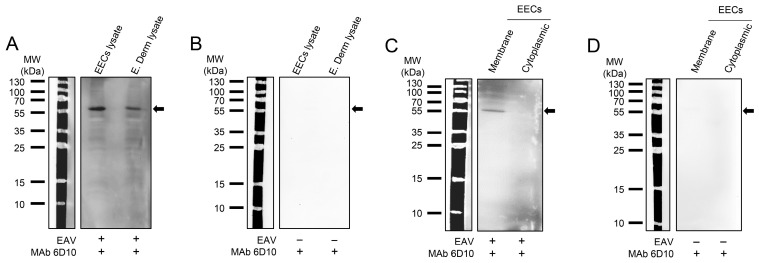
EAV interacts with a 57 kDa protein expressed on the membrane fraction of equine cells. PVDF membranes containing the protein lysates from EECs and E. Derm cells in the presence (**A**) and absence (**B**) of purified EAV. PVDF membranes containing membrane and cellular protein fractions of EECs in the presence (**C**) or absence (**D**) of purified EAV. Membranes were incubated with an anti-EAV GP5 MAb (6D10) and an HRP-conjugated anti-mouse IgG secondary antibody.

Our previous studies showed that CXCL16S protein (*CXCL16^S/S^, CXCL16^S/r^*) functions as an entry receptor for EAV [21,22]. Thus, two cell lines, EECs and E. Derm cells, susceptible to EAV infection, were first genotyped for EqCXCL16. Results show that EECs are heterozygote for CXCL16 (*CXCL16^S^*^/^*^r^*), and E. Derm cells are homozygote for the resistant allele (*CXCL16^r/r^*). The susceptibility of E. Derm cells with *CXCL16^r/r^* genotype led us to hypothesize that EAV uses different molecule(s) as entry receptors to infect E. Derm cells in vitro. To identify the EAV interacting proteins, a Far-Western blot (Far-WB) in combination with virus overlay protein binding assay (VOPBA) was performed using both EECs and E. Derm cells. Cell lysates from EECs and E. Derm cells were separated by electrophoresis through an SDS-polyacrylamide gel and transferred to a solid matrix support (PVDF membrane) before incubation with purified live EAV. The positions of the EAV binding proteins were determined after the incubation of the membrane with an anti-EAV GP5 MAb (anti-GP5, 6D10 [39]) and an HRP-conjugated anti-mouse IgG secondary antibody (Figure 1A). The results showed that both EECs and E. Derm cell lysates had a common protein with an approximate molecular weight of 57 kDa that specifically interacts with EAV. On the other hand, when the membrane was incubated with the same antibodies in the absence of purified EAV, no interacting protein was observed, confirming the specificity of the protein interacting with EAV but not with other cellular proteins (Figure 1B).

To further identify the cellular localization of this EAV binding protein, sub-cellular fractionation of EECs was performed to separate membrane and cellular proteins, and 30 µg of each fraction was separated by SDS-PAGE and transferred onto a PVDF membrane, and Far-WB coupled to VOPBA was performed. The results showed that the same 57 kDa protein that binds to EAV was present on the membrane, but not the cytoplasmic fraction of EECs (Figure 1C). When a similar membrane was incubated with the same antibodies, but in absence of purified EAV, no band was observed, confirming the specificity of the protein interacting with EAV but not with other cellular proteins (Figure 1D). Interestingly, no binding protein with a molecular weight of 30 kDa (unglycosylated CXCL16), or 35–40 kDa (glycosylated CXCL16) was observed to bind EAV. Altogether, these results show that EAV binds to a 57 kDa protein located at the membrane of the EECs and E. Derm cells.

### 3.2. Identification of Vimentin as EAV-Interacting Host Cell Protein

**Figure 2:**
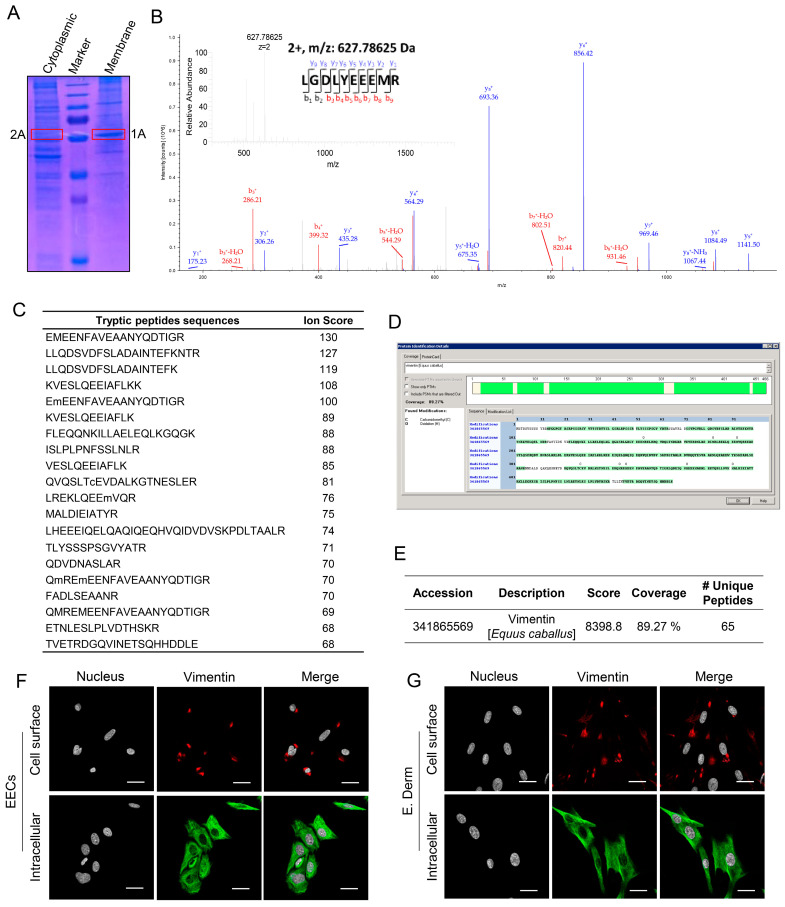
Identification and characterization of vimentin as an EAV-interacting protein. (**A**) Proteins from the cytoplasmic and membrane fractions of EECs were separated by SDS-PAGE and visualized using Coomassie Brilliant Blue staining. Band 1A (red square) in the membrane fraction corresponds to the ~57 kDa protein that was excised for mass spectrometry analysis. In contrast, no band of approximately 57 kDa was detected in the cytoplasmic fraction (2A). (**B**) LC-MS/MS analysis of the excised protein band. The MS/MS spectrum shown corresponds to a tryptic peptide, supporting identification of the protein. (**C**) Tryptic peptide sequences identified from the excised band are listed. (**D**,**E**) MASCOT search analysis of LC-MS/MS data revealed high homology to equine vimentin (Accession: 341185659), with a high Mascot score (8398.8), 89.27% sequence coverage, and 65 unique peptides identified. Immunofluorescence microscopy of EECs (**F**) and E. Derm cells (**G**) stained with anti-vimentin MAb (Clone V9) to detect cell surface cell (red) and intracellular vimentin (green). Nuclei were counterstained with DAPI (gray). Scale bars = 20 µm.

Next, to investigate EAV-interacting proteins, cytoplasmic and membrane protein fractions from EECs were separated using two denaturing polyacrylamide gels. One gel was stained with Coomassie Brilliant Blue to visualize protein bands (Figure 2A), while the other was used for Virus Overlay Protein Binding Assay (VOPBA) and Far-Western blotting to identify potential EAV-binding proteins. A distinct band of approximately 57 kDa was detected in the membrane protein fraction (1A; Figure 2A), whereas no such band was observed in the cytoplasmic fraction (2A; Figure 2A). To identify this protein, the Coomassie-stained gel of EEC membrane proteins was aligned with the VOPBA membrane, and the band corresponding to the 57 kDa region was carefully excised, processed, and analyzed by LC-MS/MS. The identity of the protein was then determined by using the homology search tool, MASCOT, where the mass/charge values were matched with the information available in the database [44]. A representative MS/MS of a selected peptide LGDLYEEEMR was shown (Figure 2B). The analysis revealed that the purified 57 kDa EAV-interacting protein showed high similarity to vimentin, with a MASCOT score of 8398.8 and a peptide sequence coverage of 89.27% (Figure 2C–E). EECs and E. Derm cells were then stained for cell surface and intracellular vimentin using an anti-vimentin MAb (Clone V9). Results show that both cell lines express not only intracellular vimentin but also cell surface vimentin at the membrane (Figure 2F,G). Altogether, these results suggest that EAV interacts specifically with the vimentin protein.

### 3.3. Vimentin Expression Is a Determinant of EAV Susceptibility

**Figure 3:**
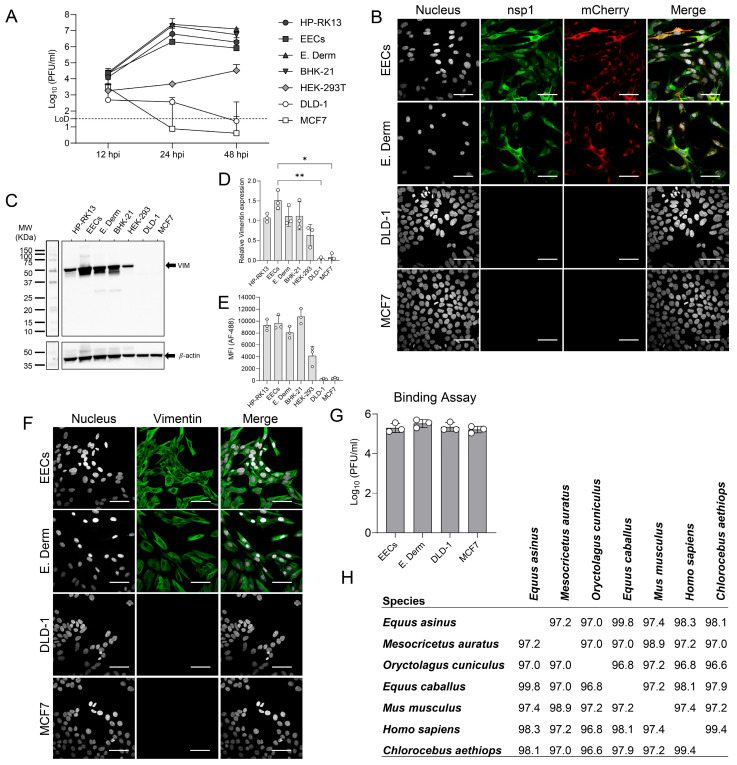
Vimentin expression is a determinant of EAV susceptibility. (**A**) EAV replication kinetics were evaluated in equine (EECs, E. Derm) and non-equine cell lines (BHK-21, HP-RK-13, HEK-293, MCF7, and DLD-1) following infection at a multiplicity of infection (MOI) of 1. (**B**) Immunofluorescence microscopy of EECs E. Derm, DLD-1, and MCF7 cells infected for 12 h with EAV sVBSmCherry and stained with EAV anti-nsp1 MAb (clone 12A4; green), to detect virus replication. Nuclei were counterstained with DAPI (gray). Scale bars = 50 µm. (**C**) Vimentin expression levels were assessed by Western blot. VIM = vimentin. (**D**) Relative quantification of vimentin expression from Western blot analysis. Vimentin levels are expressed as the ratio of each band to β-actin. (**E**) Surface expression of vimentin was quantified by flow cytometry. MFI: Mean Fluorescence Intensity. (**F**) Immunofluorescence microscopy of EECs, E. Derm, DLD-1 and MCF7 cells stained with anti-vimentin MAb (Clone V9) to detect intracellular vimentin (green). Nuclei were counterstained with DAPI (gray). Scale bars = 50 µm. (**G**) Binding assay performed on EECs, E. Derm, DLD-1, and MCF7 cells, with infectious virus titers measured 1 h post-infection at 4 °C. (**H**) Protein alignment of Vimentin from susceptible species and species where a cell line was identified as susceptible to EAV infection. Values represent the percentage of similarity. *Equus caballus*, horse, GenBank number: NP_001230074; *Equus asinus*, donkey, GenBank number: XP_014701524; *Homo sapiens*, human, GenBank number: NP_035831; *Chlorocebus aethiops*, African green monkey, GenBank number: ABA39528; *Oryctolagus cuniculus*, rabbit, GenBank number: XP_002717466; *Mus musculus*, mouse, GenBank number: NP_035831; and *Mesocricetus auratus*, hamster, GenBank number: XP_005081318. Data represent the mean ± standard deviation from three independent experiments. *, *p* < 0.05; **, *p* < 0.01.

To determine whether vimentin plays a critical role in EAV infection in vitro, cell lines derived from both horse (EECs, E. Derm) and other mammalian species (BHK-21, HP-RK-13, HEK-293, MCF7, and DLD-1) were infected with EAV sVBSmCherry at an MOI of 1, and viral replication was assessed at 12, 24, and 48 hpi through freeze–thaw lysis and subsequent virus titration. Results indicated that EECs, E. Derm, BHK-21, and RK-13 were highly susceptible to EAV infection and supported virus replication (Figure 3A). HEK-293 exhibited intermediate susceptibility with lower viral titers (≈3 Log_10_ difference at 24 hpi and ≈1.5 Log_10_ difference at 48 hpi, when compared to EECs), suggesting that HEK-293 cells restrict multiple rounds of viral replication. MCF7 and DLD-1 did not support EAV replication. Infected EECs, E. Derm, DLD-1, and MCF7 were stained for IFA at 12 hpi, allowing EAV to perform one cycle of replication (Figure 3B). High levels of nsp1 protein and mCherry were detected in both EECs and E. Derm. In contrast, neither nsp1 nor mCherry expression was observed in DLD-1 or MCF7 cells, confirming that these cell lines do not support EAV replication. Total vimentin expression was then assessed by Western blotting (Figure 3C,D), and cell surface vimentin expression was evaluated by flow cytometry (Figure 3E). High levels of total and cell surface vimentin were observed in HP-RK13, EECs, E. Derm, and BHK-21 cells, while HEK-293 cells expressed lower levels. DLD-1 and MCF7 cells showed no detectable vimentin expression (*p* = 0.008 and *p* = 0.015 for DLD-1 and MCF7 when compared to EECs, respectively) (Figure 3D). The absence of vimentin expression in DLD-1 and MCF7 was also confirmed by IFA (Figure 3F). To evaluate if vimentin expression is required for EAV to bind the target cells, a virus-binding assay was performed. EECs, E. Derm, DLD-1, and MCF7 were infected with EAV sVBSmCherry at an MOI of 1. After 1 h of adsorption at 4 °C, the cells were rinsed five times to remove unbound particles and subsequently frozen. After three cycles of freezing and thawing, bound infectious particles were titrated. The results show that EAV binds at similar levels to EECs, E. Derm, DLD-1, and MCF7 (Figure 3G), indicating that vimentin is not involved in the initial binding process but instead acts during a later stage of infection. Finally, alignment of vimentin protein sequences from susceptible equine species (*Equus caballus* and *Equus asinus*) and from susceptible cell line species (*Homo sapiens*, *Chlorocebus aethiops*, *Oryctolagus cuniculus*, *Mus musculus*, and *Mesocricetus auratus*) revealed a high degree of conservation across mammals, with sequence identity ≥96.8% (Figure 3H). These findings suggest that vimentin expression correlates with cellular susceptibility to EAV infection.

### 3.4. Pre-Treatment of EECs with Anti-Vimentin Polyclonal Antibody and Withaferin Inhibited EAV Replication

**Figure 4:**
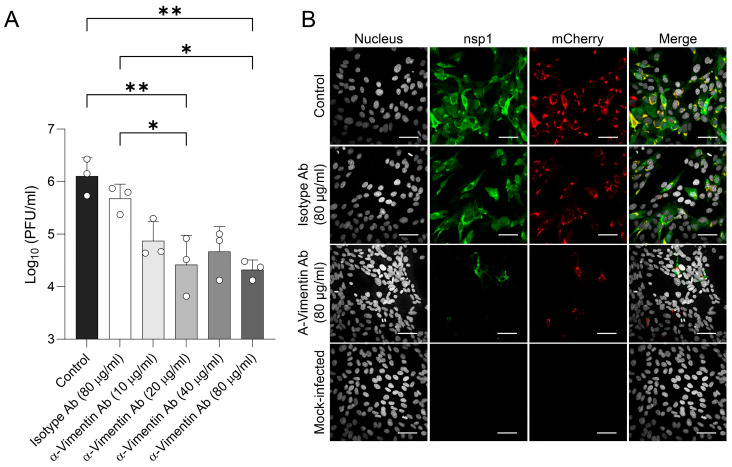
Anti-vimentin polyclonal antibody treatment partially inhibits EAV infection in EECs. (**A**) Pre-incubation of EECs with a polyclonal anti-vimentin antibody resulted in a partial reduction in infectious titers at 12 hpi, whereas an isotype control antibody had no effect. Data represent the mean ± standard deviation from three independent experiments conducted in duplicate. (**B**) Immunofluorescence microscopy of EECs pre-treated or not with isotype control and polyclonal anti-vimentin antibody (80 µg/mL) and subsequently infected for 12 h with EAV sVBSmCherry. Virus replication was visualized using EAV anti-nsp1 MAb (clone 12A4; green) and mCherry (in red). Nuclei were counterstained with DAPI (gray). Control = infected and untreated cells. Scale bars = 50 µm. *, *p* < 0.05; **, *p* < 0.01.

EECs were pre-treated with anti-vimentin polyclonal antibodies at different concentrations (10, 20, 40, and 80 μg/mL) or with an isotype control (80 μg/mL). Subsequently, cells were washed and infected with EAV at an MOI of 0.1 for 1 h at 37 °C. At 24 hpi, cells were lysed by cycles of freezing/thawing, and virus titration was performed (Figure 4A). Results showed that all the tested concentrations of anti-vimentin polyclonal antibody reduced viral titers when compared to isotype-treated cells, and a significant reduction was observed for 20 µg/mL (*p* = 0.029) and 80 µg/mL (*p* = 0.022). On the other hand, a pre-treatment with an isotype control did not show any effect (*p* > 0.05). Cells were also examined by IFA at 12 hpi (Figure 4B). High levels of nsp1 protein and mCherry were detected in both untreated EECs and those treated with 80 µg/mL of the isotype control antibody. In contrast, treatment with 80 µg/mL of the anti-vimentin polyclonal antibody resulted in only a few cells staining positive for nsp1 and mCherry. These data indicate that blocking cell surface vimentin with a polyclonal anti-vimentin antibody reduces infectious titers, supporting a role for cell surface vimentin in facilitating EAV entry in vitro.

**Figure 5:**
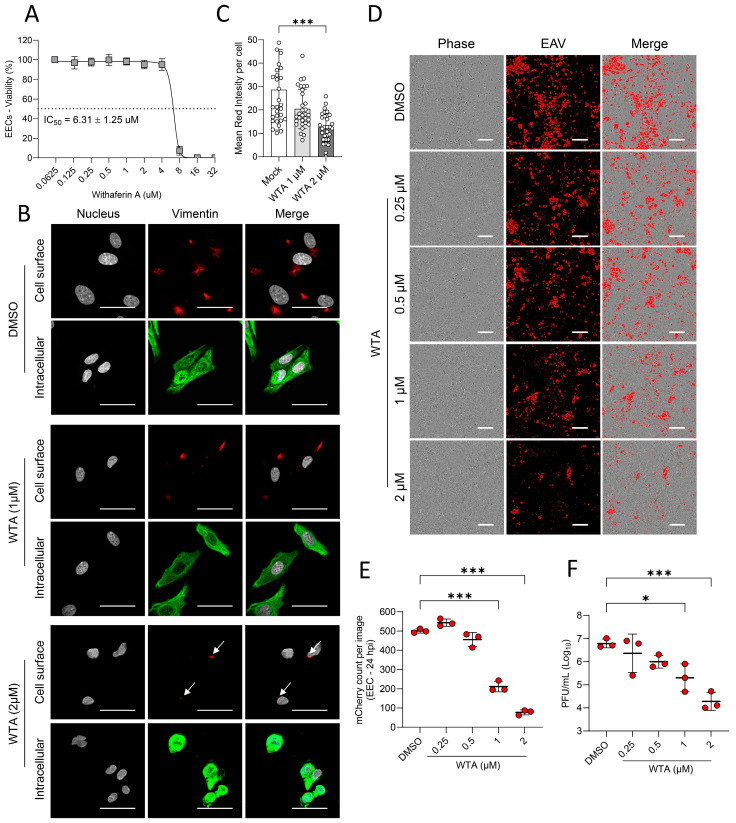
Withaferin-A pre-treatment induces a change in vimentin conformation and reduces EAV replication in EECs. (**A**) The cytotoxicity of Withaferin A (WFA) on EECs following 24 h treatment was assessed using a cell viability assay. The half-maximal inhibitory concentration (IC_50_) was calculated at 6.31 ± 1.25 µM, with significant toxicity observed at concentrations ≥ 8 µM. (**B**) Immunofluorescence analysis of vimentin in EECs treated with 0, 1, or 2 µM WFA for 6 h. Treatment with 2 µM WFA resulted in pronounced intracellular aggregation of vimentin and reduced vimentin cell surface expression, while 1 µM did not induce noticeable structural changes. The white arrows indicate a reduction in cell surface vimentin expression in the presence of 2 µM WTA. Scale = 30 µm. (**C**) Mean red fluorescence intensity was measured in 30 randomly selected EECs treated or not with 1 µM or 2 µM WTA, representing the level of cell surface vimentin expression. (**D**) Fluorescence microscopy images at 24 hpi with EAV sVBSmCherry (MOI = 0.1) following 6 h pre-treatment with indicated WFA concentrations. Reduced mCherry expression was observed at 1 and 2 µM WFA. Scale bars = 100 µm. Quantification of mCherry fluorescence intensity (**E**) and viral infectious titers in cell lysates (**F**) at 24 hpi. Both mCherry expression and virus production were significantly decreased in cells pre-treated with 1 µM and 2 µM WFA, indicating a dose-dependent inhibition of EAV replication. Data represent the mean ± standard deviation from three independent experiments conducted in duplicate. *, *p* < 0.05; *** *p*, < 0.001.

Withaferin-A (WFA), a steroidal lactone isolated from the plant *Withania somnifera*, has been shown to bind and alter the distribution of the vimentin intermediate filament in human and bovine endothelial cells in vitro [45]. Here, we evaluated the effect of WFA on vimentin conformation in EECs and its pre-treatment on EAV infection. First, a viability experiment demonstrated that WFA is highly toxic at 24 h post-treatment, starting from 8 µM and has a half maximum toxic concentration (IC_50_) of 6.31 ± 1.25 µM (Figure 5A). Immunofluorescent staining of EECs treated with WFA for 6 h demonstrated that 2 µM WFA disrupts intracellular vimentin organization, leading to its aggregation (Figure 5B) and a significant reduction in cell surface vimentin expression (*p* < 0.001; Figure 5C). At 1 µM, no reduction in cell surface vimentin expression and no aggregation of intracellular vimentin was observed (Figure 5B,C). EECs were then incubated for 6 h with different non-toxic concentrations of WFA (0.25, 0.5, 1, and 2 µM) and subsequently infected with EAV sVBSmCherry strain at an MOI of 0.1. At 24 hpi, mCherry expression was monitored using a live cell imaging microscope before cell lysis for virus titration. Results showed that 1 and 2 µM of WFA significantly reduce mCherry expression (*p* < 0.001; Figure 5D,E). Additionally, infectious titers in the cell lysate are significantly reduced following pre-treatment with 1 µM (*p* = 0.016) and 2 µM (*p* < 0.001) of WFA (Figure 5F). However, lower concentrations (0.25 µM and 0.5 µM) did not affect mCherry expression or viral titers (*p* > 0.05; Figure 5E,F). These results suggest that disruption of the vimentin’s conformation decreases the susceptibility of EECs to EAV infection.

### 3.5. Overexpression of Equine Vimentin in HEK-293 Cells Increases Cell Susceptibility to EAV

**Figure 6:**
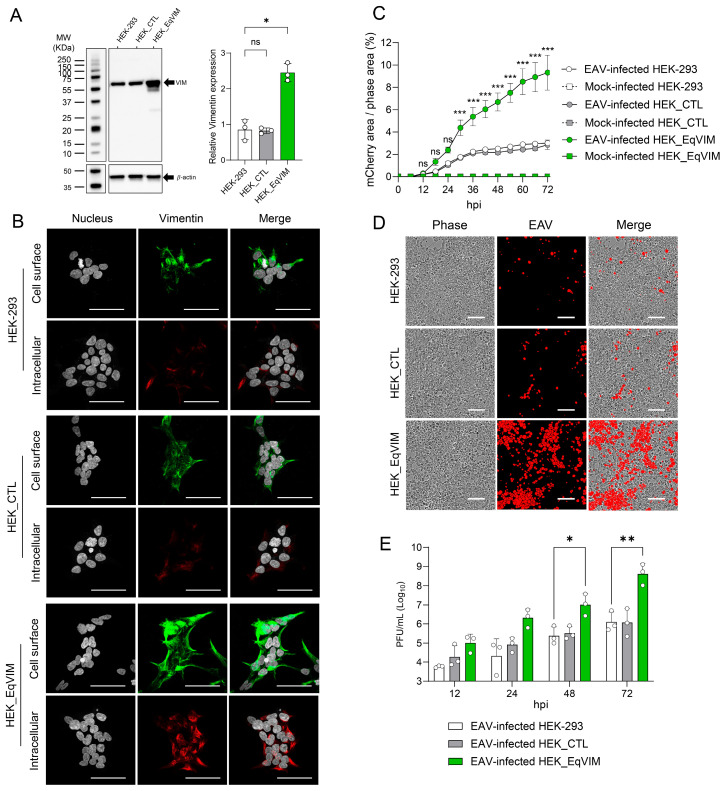
Overexpression of equine vimentin increases the susceptibility of HEK-293 cells to EAV. (**A**) Western blot analysis confirming overexpression of equine vimentin (EqVim) in stably transfected HEK-293 cells (HEK_EqVIM) compared to parental (HEK-293) and control vector-transfected cells (HEK_CTL). A significant increase in vimentin expression was observed in HEK_EqVIM cells (*p* = 0.05). (**B**) Immunofluorescence assay showing increased intracellular and cell surface vimentin staining in HEK_EqVIM cells compared to HEK-293 and HEK_CTL. (**C**) Quantification of EAV infection in HEK-293, HEK_CTL, and HEK_EqVIM cells infected with EAV sVBSmCherry (MOI = 1). The ratio of mCherry-positive area to the total cell area was monitored over 72 h by live-cell imaging. A significant increase in infection was observed in HEK_EqVIM starting from 30 hpi. Scale bars = 50 µm. (**D**) Representative fluorescence and phase contrast images at 72 hpi illustrating elevated mCherry expression in HEK_EqVIM cells compared to HEK_293. Scale bars = 100 µm. (**E**) Virus titers in culture supernatants collected at 12, 24, 48, and 72 hpi from HEK-293, HEK_CTL and HEK_EqVIM following infection at an MOI = 1. HEK_EqVIM cells produced significantly more infectious particles at 48 and 72 hpi compared to HEK_CTL. These results confirm that equine vimentin enhances susceptibility to EAV infection in vitro. Data represent the mean ± standard deviation from three independent experiments. *, *p* < 0.05; ** *p*, < 0.01; *** *p* ≤ 0.001.

To further evaluate the role of the equine vimentin protein in EAV infection, we established a stable cell line expressing the equine orthologue of vimentin (EqVim) on naïve HEK-293 cells. HEK-293 cells were transfected with either pIRESpuro3_EqVIM or with the empty control vector pIRESpuro3_CTL and cultured in the presence of 4 µg/mL puromycin. A significantly higher expression of vimentin was observed by Western blotting in the HEK_EqVIM cells, when compared to the HEK-293 (*p* = 0.05), while no difference was observed for the HEK_CTL (Figure 6A). Similarly, an increase in intracellular and cell surface vimentin expression was observed by IFA on the HEK_EqVIM cells, when compared to the HEK-293 and HEK_CTL cells (Figure 6B). HEK-293, HEK_CTL, and HEK_EqVIM cells were then infected at an MOI of 1 with EAV sVBSmCherry and incubated at 37 °C in a live cell imaging microscope. Phase and red fluorescence (mCherry expression) were recorded every 6 h for 72 h and the ratio of mCherry area (%) to the phase area (%) was graphically represented in Figure 6C. Starting from 30 hpi, this ratio was significantly higher in HEK_EqVIM infected cells when compared to HEK-293 infected cells (*p* < 0.001), whereas no significant difference was detected in HEK_CTL cells (*p* > 0.05) for all time points. This disparity is visually apparent at 72 hpi, as illustrated in Figure 6D. The cell culture supernatant was also collected at 12, 24, 48, and 72 hpi and used for virus titration (Figure 6E). A significant increase in infectious particles was observed at 48 hpi (*p* = 0.034) and 72 hpi (*p* = 0.008) in the cell culture supernatant of HEK_EqVIM when compared to HEK-293, while no significant difference was observed for the HEK_CTL (*p* > 0.05). Concordant with the previous finding, this result shows that the presence of equine vimentin increases the susceptibility to EAV infection, confirming its important role in EAV infection in vitro.

## 4. Discussion

In previous studies, we have shown that a susceptible allelic variant of equine transmembrane CXCL16 (CXCL16S [*EqCXCL16^S/S^* or *EqCXCl16^S/r^*]) acts as an entry receptor for EAV by binding with the virus and facilitating its internalization inside the host cells [21,22]. This susceptibility phenotype is associated with four non-synonymous mutations located in exon 1 of the EqCXCL16 gene. In contrast, the resistant variant, CXCL16R, found in horses lacking these mutations (EqCXCL16*^r/r^*), fails to bind EAV in vitro [22]. However, cells such as CD14+ monocytes from *EqCXCL16^r/r^* horses and E. Derm, expressing only EqCXCL16R, are also susceptible to EAV infection. This led us to hypothesize that other host cell factors are involved in EAV infection. Recent work conducted by others identified CD81 and neonatal Fc receptor (FcRn) as EAV receptors [31,46].

Here, we demonstrated that vimentin, a type III intermediate filament protein [47,48], plays a determinant role in EAV infection. Vimentin has a molecular weight of 57 kDa and is expressed in various cells of mesenchymal origin, such as T lymphocytes, monocytes, fibroblasts, and endothelial cells [49]. Besides its structural role, vimentin may also be expressed at the cell surface [50,51]. Cell surface vimentin was reported as an attachment factor or co-receptor for various viruses belonging to the *Nidovirales* order, such as the related Porcine Reproductive and Respiratory Virus (PRRSV) [52], SARS-CoV [53] and SARS-CoV-2 [54,55], but also for viruses belonging to more distant orders, such as Zika virus [56], Dengue virus [57], Human Papillomavirus [58], Japanese Encephalitis virus [59,60], and Enterovirus 71 [61]. In this study, cell surface equine vimentin was identified to play a role in the attachment and infection of EAV. We first identified a 57 kDa protein expressed in the cell membrane of EECs as a receptor for EAV, which was further identified as vimentin by LC-MS/MS analysis. Consistent with our previous findings showing that EAV infects and persists in the stallion ampullae with a specific tropism for vimentin-positive stromal cells in the lamina propria, these results further support vimentin as a key host factor in EAV infection and persistence, potentially contributing to the maintenance of viral reservoirs in the reproductive tract [13]. Vimentin plays a multifaceted role in PRRSV infection, acting as both an attachment factor and an intracellular replication cofactor through interactions with the viral nucleocapsid and non-structural proteins [52,62,63]. Moreover, vimentin rearranges into cage-like structures around PRRSV replication complexes, supporting viral replication and suggesting a conserved role for vimentin in arterivirus persistence [64].

Surprisingly, no other EAV-binding proteins, such as EqCXCL16S, CD81, or FcRn, were detected by Far-Western blot in our assay, suggesting that vimentin may play a more prominent or direct role in viral attachment under these experimental conditions.

A clear correlation was observed between vimentin expression and cellular susceptibility to EAV infection. Although vimentin is highly conserved among mammalian species, EAV infection in vivo remains species-specific and restricted to equids, suggesting that its conserved nature primarily reflects a basic cellular role as an attachment factor rather than implying that EAV can utilize vimentin from other species as an entry receptor. In this study, we identified two cell lines, DLD-1 and MCF7, resistant to EAV infection. Notably, these cell lines do not express vimentin, as previously reported [65,66]. However, it is important to note that EAV can bind to DLD-1 and MCF7 at a similar level to EECs and E. Derm, demonstrating that vimentin is not involved in the initial binding process but instead acts during a later stage of infection (e.g., viral internalization or membrane fusion).

HEK-293 cells, which naturally express low levels of vimentin [67], supported only limited EAV replication. Overexpression of equine vimentin in these cells (HEK_EqVIM) significantly enhanced their susceptibility to infection, supporting the role of vimentin as a key attachment factor facilitating EAV entry. Overall, these findings support the role of vimentin as a key attachment factor facilitating EAV entry in vitro. While equine vimentin enhances susceptibility in heterologous cells, we have not directly shown that its expression alone is sufficient to confer permissiveness in vimentin-negative cells. Attempts to establish stable EqVIM expression in MCF7 and DLD-1 cells were unsuccessful. Alternative expression strategies, such as retroviral vector-mediated delivery, may be explored in future studies. Attempts to generate vimentin knockouts in EECs and E. Derm cells were unsuccessful due to cytotoxicity, highlighting the essential role of vimentin in maintaining cellular morphology. Alternative strategies, such as conditional knockdown, transient silencing, or the use of dominant-negative mutants, may help overcome this limitation and further clarify vimentin’s role in EAV infection.

Blocking the cell surface vimentin domain with vimentin polyclonal antibody decreases the early replication cycle of EAV. A similar observation was made when EECs were pre-treated with WFA, which caused vimentin conformational disruption, aggregation, and a reduction in cell surface vimentin expression. While the primary function of WFA is the alteration of vimentin conformation [45,68], it was also reported that WFA does not prevent the attachment and entry of Chikungunya virus, but acts as a viral protease (nsp2) inhibitor [69]. Therefore, while in line with the results observed with the vimentin polyclonal antibody, we cannot conclude that the decrease in viral replication and progeny virus production after WFA treatment is solely due to inhibition of cell surface vimentin expression. Further studies, such as assessing whether WFA interacts with EAV nsp2 or other viral proteins, will be required to clarify its mechanism of action.

Altogether, our findings indicate that cell surface equine vimentin plays a critical role in EAV infection in vitro and may function as an important attachment cofactor facilitating viral entry. Although the precise mechanism remains unclear, our data, together with prior studies on PRRSV and SARS-CoV-2 [52,54,70], suggest that vimentin may not act as a direct binding receptor but rather as a scaffold or accessory factor that stabilizes or enhances interactions between EAV and its primary receptors. Such receptors may include EqCXCL16S, CD81, FcRn, heparan sulfate, or other yet-unidentified surface molecules. Further studies, including co-immunoprecipitation and domain-mapping approaches, will be necessary to identify the vimentin domain(s) that interact with EAV, to determine whether vimentin directly engages EAV structural proteins, and to investigate its potential role in modulating the availability or function of the EAV receptor. In particular, analyses in E. derm cells could help clarify the interaction between vimentin and the yet-to-be-characterized EAV receptor.

## Data Availability

The data presented in this study are available on request from the corresponding author.

## Abbreviations

The following abbreviations are used in this manuscript:

AF	Alexa Fluor
BHK-21	Baby Hamster Kidney fibroblasts
CID	Collision Induced Dissociation
CTL	Control
CXCL16	C-X-C motif chemokine ligand 16
DLD-1	Human colorectal adenocarcinoma cell line
DMEM	Dulbecco’s Modified Eagle’s Medium
DTT	Dithiothreitol
E. Derms	Equine Dermal cells
EAV	Equine arteritis virus
EDTA	Ethylenediaminetetraacetic Acid
EECs	Equine Pulmonary Artery Endothelial Cells
EMEM	Eagle’s Minimal Essential Medium
EqVIM	Equine Vimentin
EVA	Equine Viral Arteritis
FBS	Fetal Bovine Serum
FcRn	Neonatal Fc receptor
FDRs	False Discovery Rates
GP5	Glycoprotein 5
hpi	Hours post-infection
HRP	Horseradish Peroxidase
IC_50_	Half Maximum Toxic Concentration
IFA	Immunofluorescence Assay
IgG	Immunoglobulin G
IU	International Unit
kDa	Kilodaltons
LC-MS/MS	Liquid Chromatography-tandem Mass Spectrometry
L-M	mouse connective tissue cells
LTPI	Long-Term Persistent Infection
Mab	Monoclonal Antibody
MCF7	Human breast cancer cell line
MOI	Multiplicity Of Infection
N	Nucleocapsid
NCBI	National Center for Biotechnology Information
PBMCs	Peripheral Blood Mononuclear Cells
PBS	Phosphate-Buffered Saline
PFA PFU	Paraformaldehyde Plaque Forming Unit
PMSF	Phenylmethylsulfonyl Fluoride
PVDF	Polyvinylidene Fluoride
RK-13	normal Rabbit Kidney epithelial cells
RT	Room Temperature
*v*/*v*	Volume/Volume
VBS	Virulent Bucyrus Strain
VOPBA	Virus Overlay Protein Binding Assay
WB	Western-Blot
WFA	Withaferin A

## References

[1] Kuhn J.H., Lauck M., Bailey A.L., Shchetinin A.M., Vishnevskaya T.V., Bào Y., Ng T.F.F., LeBreton M., Schneider B.S., Gillis A. (2016). Reorganization and Expansion of the Nidoviral Family Arteriviridae. Arch. Virol..

[2] Brinton M.A., Gulyaeva A.A., Balasuriya U.B.R., Dunowska M., Faaberg K.S., Goldberg T., Leung F.C.C., Nauwynck H.J., Snijder E.J., Stadejek T. (2021). ICTV Virus Taxonomy Profile: Arteriviridae 2021. J. Gen. Virol..

[3] Bryans J.T., Crowe M.E., Doll E.R., McCollum W.H. (1957). Isolation of a Filterable Agent Causing Arteritis of Horses and Abortion by Mares; Its Differentiation from the Equine Abortion (Influenza) Virus. Cornell Vet..

[4] Doll E.R., Knappenberger R.E., Bryans J.T. (1957). An Outbreak of Abortion Caused by the Equine Arteritis Virus. Cornell Vet..

[5] Balasuriya U.B.R., Carossino M., Timoney P.J. (2018). Equine Viral Arteritis: A Respiratory and Reproductive Disease of Significant Economic Importance to the Equine Industry. Equine Vet. Educ..

[6] Timoney P.J., McCollum W.H. (1993). Equine Viral Arteritis. Vet. Clin. N. Am. Equine Pract..

[7] Balasuriya U.B.R., Snijder E.J., Heidner H.W., Zhang J., Zevenhoven-Dobbe J.C., Boone J.D., McCollum W.H., Timoney P.J., MacLachlan N.J. (2007). Development and Characterization of an Infectious cDNA Clone of the Virulent Bucyrus Strain of Equine Arteritis Virus. J. Gen. Virol..

[8] Balasuriya U.B., Snijder E.J., van Dinten L.C., Heidner H.W., Wilson W.D., Hedges J.F., Hullinger P.J., MacLachlan N.J. (1999). Equine Arteritis Virus Derived from an Infectious cDNA Clone Is Attenuated and Genetically Stable in Infected Stallions. Virology.

[9] Balasuriya U.B.R., Go Y.Y., MacLachlan N.J. (2013). Equine Arteritis Virus. Vet. Microbiol..

[10] Balasuriya U.B.R. (2014). Equine Viral Arteritis. Vet. Clin. N. Am. Equine Pract..

[11] Campos J.R., Breheny P., Araujo R.R., Troedsson M.H.T., Squires E.L., Timoney P.J., Balasuriya U.B.R. (2014). Semen Quality of Stallions Challenged with the Kentucky 84 Strain of Equine Arteritis Virus. Theriogenology.

[12] Vairo S., Vandekerckhove A., Steukers L., Glorieux S., Van den Broeck W., Nauwynck H. (2012). Clinical and Virological Outcome of an Infection with the Belgian Equine Arteritis Virus Strain 08P178. Vet. Microbiol..

[13] Carossino M., Loynachan A.T., Canisso I.F., Cook R.F., Campos J.R., Nam B., Go Y.Y., Squires E.L., Troedsson M.H.T., Swerczek T. (2017). Equine Arteritis Virus Has Specific Tropism for Stromal Cells and CD8 ^+^ T and CD21 ^+^ B Lymphocytes but Not for Glandular Epithelium at the Primary Site of Persistent Infection in the Stallion Reproductive Tract. J. Virol..

[14] Cole J.R., Hall R.F., Gosser H.S., Hendricks J.B., Pursell A.R., Senne D.A., Pearson J.E., Gipson C.A. (1986). Transmissibility and Abortogenic Effect of Equine Viral Arteritis in Mares. J. Am. Vet. Med. Assoc..

[15] Vaala W.E., Hamir A.N., Dubovi E.J., Timoney P.J., Ruiz B. (1992). Fatal, Congenitally Acquired Infection with Equine Arteritis Virus in a Neonatal Thoroughbred. Equine Vet. J..

[16] Carossino M., Dini P., Kalbfleisch T.S., Loynachan A.T., Canisso I.F., Cook R.F., Timoney P.J., Balasuriya U.B.R. (2019). Equine Arteritis Virus Long-Term Persistence Is Orchestrated by CD8+ T Lymphocyte Transcription Factors, Inhibitory Receptors, and the CXCL16/CXCR6 Axis. PLoS Pathog..

[17] Carossino M., Wagner B., Loynachan A.T., Cook R.F., Canisso I.F., Chelvarajan L., Edwards C.L., Nam B., Timoney J.F., Timoney P.J. (2017). Equine Arteritis Virus Elicits a Mucosal Antibody Response in the Reproductive Tract of Persistently Infected Stallions. Clin. Vaccine Immunol..

[18] Go Y.Y., Zhang J., Timoney P.J., Cook R.F., Horohov D.W., Balasuriya U.B.R. (2010). Complex Interactions between the Major and Minor Envelope Proteins of Equine Arteritis Virus Determine Its Tropism for Equine CD3 ^+^ T Lymphocytes and CD14 ^+^ Monocytes. J. Virol..

[19] Go Y.Y., Bailey E., Cook D.G., Coleman S.J., MacLeod J.N., Chen K.-C., Timoney P.J., Balasuriya U.B.R. (2011). Genome-Wide Association Study among Four Horse Breeds Identifies a Common Haplotype Associated with In Vitro CD3+ T Cell Susceptibility/Resistance to Equine Arteritis Virus Infection. J. Virol..

[20] Go Y.Y., Bailey E., Timoney P.J., Shuck K.M., Balasuriya U.B.R. (2012). Evidence That In Vitro Susceptibility of CD3+ T Lymphocytes to Equine Arteritis Virus Infection Reflects Genetic Predisposition of Naturally Infected Stallions To Become Carriers of the Virus. J. Virol..

[21] Sarkar S., Chelvarajan L., Go Y.Y., Cook F., Artiushin S., Mondal S., Anderson K., Eberth J., Timoney P.J., Kalbfleisch T.S. (2016). Equine Arteritis Virus Uses Equine CXCL16 as an Entry Receptor. J. Virol..

[22] Sarkar S., Bailey E., Go Y.Y., Cook R.F., Kalbfleisch T., Eberth J., Chelvarajan R.L., Shuck K.M., Artiushin S., Timoney P.J. (2016). Allelic Variation in CXCL16 Determines CD3+ T Lymphocyte Susceptibility to Equine Arteritis Virus Infection and Establishment of Long-Term Carrier State in the Stallion. PLoS Genet..

[23] Doll E.R., Bryans J.T., Wilson J.C. (1968). Immunization against Equine Viral Arteritis Using Modified Live Virus Propagated in Cell Cultures of Rabbit Kidney. Cornell Vet..

[24] Radwan A.I., Burger D. (1973). The Complement-Requiring Neutralization of Equine Arteritis Virus by Late Antisera. Virology.

[25] Konishi S., Akashi H., Sentsui H., Ogata M. (1975). Studies on Equine Viral Arteritis I. Characterization of the Virus and Trial Survey on Antibody with Vero Cell Cultures. Jap. J. Vet. Sci..

[26] Harry T.O., McCollum W.H. (1981). Stability of Viability and Immunizing Potency of Lyophilized, Modified Equine Arteritis Live-Virus Vaccine. Am. J. Vet. Res..

[27] Van Berlo M.F., Horzinek M.C., Van der Zeijst B.A.M. (1982). Equine Arteritis Virus-Infected Cells Contain Six Polyadenylated Virus-Specific RNAs. Virology.

[28] Zhang J., Timoney P.J., MacLachlan N.J., McCollum W.H., Balasuriya U.B.R. (2008). Persistent Equine Arteritis Virus Infection in HeLa Cells. J. Virol..

[29] Lu Z., Zhang J., Huang C.M., Go Y.Y., Faaberg K.S., Rowland R.R.R., Timoney P.J., Balasuriya U.B.R. (2012). Chimeric Viruses Containing the N-Terminal Ectodomains of GP5 and M Proteins of Porcine Reproductive and Respiratory Syndrome Virus Do Not Change the Cellular Tropism of Equine Arteritis Virus. Virology.

[30] Metz G., Abeyá M., Serena M., Panei C., Echeverría M. (2019). Evaluation of Apoptosis Markers in Different Cell Lines Infected with Equine Arteritis Virus. Biotech. Histochem..

[31] Maloney S.M., Shaw T.M., Nennig K.M., Larsen M.S., Shah A., Kumar A., Marcotrigiano J., Grove J., Snijder E.J., Kirchdoerfer R.N. (2025). CD81 Is a Receptor for Equine Arteritis Virus (Family: Arteriviridae). mBio.

[32] Asagoe T., Inaba Y., Jusa E.R., Kouno M., Uwatoko K., Fukunaga Y. (1997). Effect of Heparin on Infection of Cells by Equine Arteritis Virus. J. Vet. Med. Sci..

[33] Lu Z., Sarkar S., Zhang J., Balasuriya U.B.R. (2016). Conserved Arginine Residues in the Carboxyl Terminus of the Equine Arteritis Virus E Protein May Play a Role in Heparin Binding but May Not Affect Viral Infectivity in Equine Endothelial Cells. Arch. Virol..

[34] Hedges J.F., Demaula C.D., Moore B.D., Mclaughlin B.E., Simon S.I., Maclachlan N.J. (2001). Characterization of Equine E-Selectin. Immunology.

[35] Moore B.D., Balasuriya U.B.R., Hedges J.F., MacLachlan N.J. (2002). Growth Characteristics of a Highly Virulent, a Moderately Virulent, and an Avirulent Strain of Equine Arteritis Virus in Primary Equine Endothelial Cells Are Predictive of Their Virulence to Horses. Virology.

[36] Go Y.Y., Li Y., Chen Z., Han M., Yoo D., Fang Y., Balasuriya U.B.R. (2014). Equine Arteritis Virus Does Not Induce Interferon Production in Equine Endothelial Cells: Identification of Nonstructural Protein 1 as a Main Interferon Antagonist. BioMed Res. Int..

[37] Mondal S.P., Cook R.F., Chelvarajan R.L., Henney P.J., Timoney P.J., Balasuriya U.B.R. (2016). Development and Characterization of a Synthetic Infectious cDNA Clone of the Virulent Bucyrus Strain of Equine Arteritis Virus Expressing mCherry (Red Fluorescent Protein). Arch. Virol..

[38] Wagner H.M., Balasuriya U.B.R., James MacLachlan N. (2003). The Serologic Response of Horses to Equine Arteritis Virus as Determined by Competitive Enzyme-Linked Immunosorbent Assays (c-ELISAs) to Structural and Non-Structural Viral Proteins. Comp. Immunol. Microbiol. Infect. Dis..

[39] Balasuriya U.B., Rossitto P.V., DeMaula C.D., MacLachlan N.J. (1993). A 29K Envelope Glycoprotein of Equine Arteritis Virus Expresses Neutralization Determinants Recognized by Murine Monoclonal Antibodies. J. Gen. Virol..

[40] Yang L., Gal J., Chen J., Zhu H. (2014). Self-Assembled FUS Binds Active Chromatin and Regulates Gene Transcription. Proc. Natl. Acad. Sci. USA.

[41] Thieulent C., Hue E.S., Sutton G., Fortier C., Dallemagne P., Zientara S., Munier-Lehmann H., Hans A., Paillot R., Vidalain P.-O. (2020). Identification of Antiviral Compounds against Equid Herpesvirus-1 Using Real-Time Cell Assay Screening: Efficacy of Decitabine and Valganciclovir Alone or in Combination. Antivir. Res..

[42] Sarkar S., Balasuriya U.B.R., Horohov D.W., Chambers T.M. (2016). The Neuropathogenic T953 Strain of Equine Herpesvirus-1 Inhibits Type-I IFN Mediated Antiviral Activity in Equine Endothelial Cells. Vet. Microbiol..

[43] Thieulent C.J., Carossino M., Balasuriya U.B.R., Graves K., Bailey E., Eberth J., Canisso I.F., Andrews F.M., Keowen M.L., Go Y.Y. (2022). Development of a TaqMan® Allelic Discrimination qPCR Assay for Rapid Detection of Equine CXCL16 Allelic Variants Associated with the Establishment of Long-Term Equine Arteritis Virus Carrier State in Stallions. Front. Genet..

[44] Perkins D.N., Pappin D.J.C., Creasy D.M., Cottrell J.S. (1999). Probability-Based Protein Identification by Searching Sequence Databases Using Mass Spectrometry Data. Electrophoresis.

[45] Bargagna-Mohan P., Hamza A., Kim Y., Khuan (Abby) Ho Y., Mor-Vaknin N., Wendschlag N., Liu J., Evans R.M., Markovitz D.M., Zhan C.-G. (2007). The Tumor Inhibitor and Antiangiogenic Agent Withaferin A Targets the Intermediate Filament Protein Vimentin. Chem. Biol..

[46] Shaw T.M., Huey D., Mousa-Makky M., Compaleo J., Nennig K., Shah A.P., Jiang F., Qiu X., Klipsic D., Rowland R.R.R. (2024). The Neonatal Fc Receptor (FcRn) Is a Pan-Arterivirus Receptor. Nat. Commun..

[47] Dutour-Provenzano G., Etienne-Manneville S. (2021). Intermediate Filaments. Curr. Biol..

[48] Arrindell J., Desnues B. (2023). Vimentin: From a Cytoskeletal Protein to a Critical Modulator of Immune Response and a Target for Infection. Front. Immunol..

[49] Ramos I., Stamatakis K., Oeste C.L., Pérez-Sala D. (2020). Vimentin as a Multifaceted Player and Potential Therapeutic Target in Viral Infections. Int. J. Mol. Sci..

[50] Mor-Vaknin N., Punturieri A., Sitwala K., Markovitz D.M. (2003). Vimentin Is Secreted by Activated Macrophages. Nat. Cell Biol..

[51] Päll T., Pink A., Kasak L., Turkina M., Anderson W., Valkna A., Kogerman P. (2011). Soluble CD44 Interacts with Intermediate Filament Protein Vimentin on Endothelial Cell Surface. PLoS ONE.

[52] Kim J.-K., Fahad A.-M., Shanmukhappa K., Kapil S. (2006). Defining the Cellular Target(s) of Porcine Reproductive and Respiratory Syndrome Virus Blocking Monoclonal Antibody 7G10. J. Virol..

[53] Yu Y.T.-C., Chien S.-C., Chen I.-Y., Lai C.-T., Tsay Y.-G., Chang S.C., Chang M.-F. (2016). Surface Vimentin Is Critical for the Cell Entry of SARS-CoV. J. Biomed. Sci..

[54] Amraei R., Xia C., Olejnik J., White M.R., Napoleon M.A., Lotfollahzadeh S., Hauser B.M., Schmidt A.G., Chitalia V., Mühlberger E. (2022). Extracellular Vimentin Is an Attachment Factor That Facilitates SARS-CoV-2 Entry into Human Endothelial Cells. Proc. Natl. Acad. Sci. USA.

[55] Lalioti V., González-Sanz S., Lois-Bermejo I., González-Jiménez P., Viedma-Poyatos Á., Merino A., Pajares M.A., Pérez-Sala D. (2022). Cell Surface Detection of Vimentin, ACE2 and SARS-CoV-2 Spike Proteins Reveals Selective Colocalization at Primary Cilia. Sci. Rep..

[56] Zhang Y., Zhao S., Li Y., Feng F., Li M., Xue Y., Cui J., Xu T., Jin X., Jiu Y. (2022). Host Cytoskeletal Vimentin Serves as a Structural Organizer and an RNA-Binding Protein Regulator to Facilitate Zika Viral Replication. Proc. Natl. Acad. Sci. USA.

[57] Yang J., Zou L., Yang Y., Yuan J., Hu Z., Liu H., Peng H., Shang W., Zhang X., Zhu J. (2016). Superficial Vimentin Mediates DENV-2 Infection of Vascular Endothelial Cells. Sci. Rep..

[58] Schäfer G., Graham L.M., Lang D.M., Blumenthal M.J., Bergant Marušič M., Katz A.A. (2017). Vimentin Modulates Infectious Internalization of Human Papillomavirus 16 Pseudovirions. J. Virol..

[59] Das S., Ravi V., Desai A. (2011). Japanese Encephalitis Virus Interacts with Vimentin to Facilitate Its Entry into Porcine Kidney Cell Line. Virus Res..

[60] Liang J.-J., Yu C.-Y., Liao C.-L., Lin Y.-L. (2011). Vimentin Binding Is Critical for Infection by the Virulent Strain of Japanese Encephalitis Virus: Virulent JEV Binds Vimentin for Infection. Cell. Microbiol..

[61] Du N., Cong H., Tian H., Zhang H., Zhang W., Song L., Tien P. (2014). Cell Surface Vimentin Is an Attachment Receptor for Enterovirus 71. J. Virol..

[62] Song T., Fang L., Wang D., Zhang R., Zeng S., An K., Chen H., Xiao S. (2016). Quantitative Interactome Reveals That Porcine Reproductive and Respiratory Syndrome Virus Nonstructural Protein 2 Forms a Complex with Viral Nucleocapsid Protein and Cellular Vimentin. J. Proteom..

[63] Liang Z., Li P., Wang C., Singh D., Zhang X. (2020). Visualizing the Transport of Porcine Reproductive and Respiratory Syndrome Virus in Live Cells by Quantum Dots-Based Single Virus Tracking. Virol. Sin..

[64] Zheng X., Li R., Qiao S., Chen X., Zhang L., Lu Q., Xing G., Zhou E., Zhang G. (2021). Vimentin Rearrangement by Phosphorylation Is Beneficial for Porcine Reproductive and Respiratory Syndrome Virus Replication In Vitro. Vet. Microbiol..

[65] Tanaka S., Kobayashi W., Haraguchi M., Ishihata K., Nakamura N., Ozawa M. (2016). Snail1 Expression in Human Colon Cancer DLD-1 Cells Confers Invasive Properties without N-Cadherin Expression. Biochem. Biophys. Rep..

[66] Liu C.-Y., Lin H.-H., Tang M.-J., Wang Y.-K. (2015). Vimentin Contributes to Epithelial-Mesenchymal Transition Cancer Cell Mechanics by Mediating Cytoskeletal Organization and Focal Adhesion Maturation. Oncotarget.

[67] Inada M., Izawa G., Kobayashi W., Ozawa M. (2016). 293 Cells Express Both Epithelial as Well as Mesenchymal Cell Adhesion Molecules. Int. J. Mol. Med..

[68] Grin B., Mahammad S., Wedig T., Cleland M.M., Tsai L., Herrmann H., Goldman R.D. (2012). Withaferin A Alters Intermediate Filament Organization, Cell Shape and Behavior. PLoS ONE.

[69] Sharma K.B., Subramani C., Ganesh K., Sharma A., Basu B., Balyan S., Sharma G., Ka S., Deb A., Srivastava M. (2024). Withaferin A Inhibits Chikungunya Virus nsP2 Protease and Shows Antiviral Activity in the Cell Culture and Mouse Model of Virus Infection. PLoS Pathog..

[70] Arrindell J., Abou Atmeh P., Jayet L., Sereme Y., Mege J.-L., Desnues B. (2022). Vimentin Is an Important ACE2 Co-Receptor for SARS-CoV-2 in Epithelial Cells. iScience.

